# From Global to Local—New Insights into Features of Pyrethroid Detoxification in Vector Mosquitoes

**DOI:** 10.3390/insects12040276

**Published:** 2021-03-24

**Authors:** William C. Black, Trey K. Snell, Karla Saavedra-Rodriguez, Rebekah C. Kading, Corey L. Campbell

**Affiliations:** Department of Microbiology, Immunology and Pathology, Colorado State University, Fort Collins, CO 80523, USA; william.black@colostate.edu (W.C.B.IV); tksnell@rams.colostate.edu (T.K.S.); karla.saavedra_rodriguez@colostate.edu (K.S.-R.); rebekah.kading@colostate.edu (R.C.K.)

**Keywords:** metabolic resistance, insecticide, detoxification, pyrethroid, deltamethrin, permethrin, mosquito, *Anopheles*, *Aedes*, *Culex*

## Abstract

**Simple Summary:**

Insecticides are used to reduce the nuisance of biting insects, and, importantly, also to reduce the spread of insect-borne diseases. Over the years, mosquitoes have developed resistance to these insecticides. The most commonly used class of insecticides are the pyrethroids, synthetic versions of plant-derived compounds; they are less toxic to mammals and other animals than some other classes. One problem with all insecticides, though, is the development of resistance by the insect groups they are applied to combat. This review describes new insights into the ways in which mosquitoes have evolved resistance to pyrethroids. For example, before pyrethroids bind to their targets on motoneurons to paralyze mosquitoes, they must first pass through the outer exoskeleton to inner tissues. Resistant mosquitoes have evolved the ability to break down pyrethroids into nontoxic products that are then excreted. This metabolism prevents toxic buildup of the insecticide, which would otherwise be lethal to the mosquitoes. Scientists have identified a variety of changes to mosquito genes that are responsible for insecticide degradation and excretion. In this review, we outline the genes and pathways involved in the breakdown of pyrethroids and the key gene categories that are involved.

**Abstract:**

The threat of mosquito-borne diseases continues to be a problem for public health in subtropical and tropical regions of the world; in response, there has been increased use of adulticidal insecticides, such as pyrethroids, in human habitation areas over the last thirty years. As a result, the prevalence of pyrethroid-resistant genetic markers in natural mosquito populations has increased at an alarming rate. This review details recent advances in the understanding of specific mechanisms associated with pyrethroid resistance, with emphasis on features of insecticide detoxification and the interdependence of multiple cellular pathways. Together, these advances add important context to the understanding of the processes that are selected in resistant mosquitoes. Specifically, before pyrethroids bind to their targets on motoneurons, they must first permeate the outer cuticle and diffuse to inner tissues. Resistant mosquitoes have evolved detoxification mechanisms that rely on cytochrome P450s (CYP), esterases, carboxyesterases, and other oxidation/reduction (redox) components to effectively detoxify pyrethroids to nontoxic breakdown products that are then excreted. Enhanced resistance mechanisms have evolved to include alteration of gene copy number, transcriptional and post-transcriptional regulation of gene expression, as well as changes to cellular signaling mechanisms. Here, we outline the variety of ways in which detoxification has been selected in various mosquito populations, as well as key gene categories involved. Pathways associated with potential new genes of interest are proposed. Consideration of multiple cellular pathways could provide opportunities for development of new insecticides.

## 1. Introduction

The global burden of mosquito-borne diseases on human health continues to grow (reviewed in [1]). Pyrethroids, such as permethrin, deltamethrin, and similar derivatives, are the primary compounds used to control adult mosquito populations near human dwellings (reviewed in [2]). In areas where adulticides were applied to protect people from the nuisance of mosquitoes and spread of arboviral and parasitic pathogens, a sharp rise in resistant mosquitoes has occurred [3,4,5,6,7,8]. Mosquitoes have adapted in a variety of ways to alleviate or bypass the effects of pyrethroid toxicity through genetic, structural, and physiological mechanisms. Target site resistance, which was first described by Busvine for houseflies in the early 1950s [9], initially occurred following widespread use of dichloro-diphenyl-trichloroethane (DDT) in the early 20th century and was later shown to co-occur with resistance to pyrethroids [10]. The resistance locus was eventually ascribed to the pyrethroid target, the voltage-gated sodium channel (VGSC, PARA, NAV), which is located on motoneurons [11]. Additional major adaptive features occur in biochemical resistance or detoxification mechanisms [12,13,14,15,16,17]. Moreover, thickening of exoskeleton cuticle [18,19,20,21,22] has also been selected. Together, these responses comprise the mechanisms of physiological resistance. Genes and cellular components involved in pyrethroid inactivation or clearance are often characterized by assessing changes in the toxicological phenotypes of paralysis, or knockdown, and lethality [7,23,24,25]. 

Here, we detail major advances and new insights into various resistance mechanisms, with focus on the molecular features of resistance genes, specifically genomic structural changes, and how resistance is controlled by upstream regulatory processes. Because target site mutations at VGSC have been the subject of numerous studies [11,26,27,28,29], here, VGSC is covered in the context of intersections with other resistance or regulatory mechanisms. For a detailed review of target site mutations, see Du et al. [30]. Characteristics of metabolic resistance and other features, with particular focus on recent functional studies and key genes involved in biochemical, post-transcriptional, and cell signaling mechanisms, will be explored. This review is not exhaustive; rather, we focus on highlights of mechanistic features of pyrethroid resistance or those that contribute to a broader understanding of the systemic effects of selection. 

### Multiple Pathways Contribute to Resistance Phenotypes

**Figure 1:**
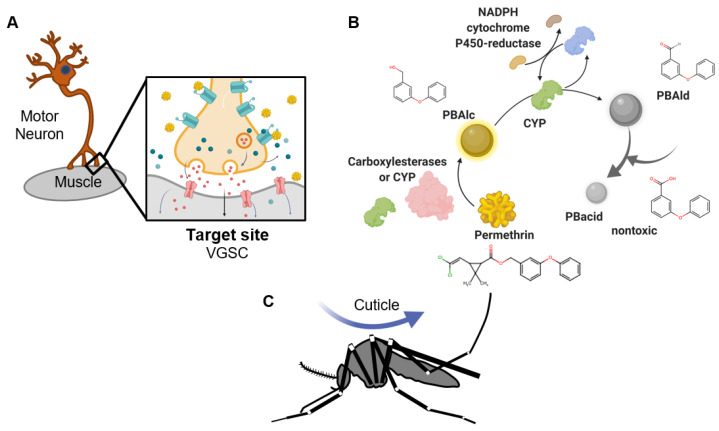
Three major pyrethroid resistance mechanisms. (**A**) At the target site, pyrethroid molecules (gold spheres) bind voltage-gated sodium channel (VGSC) (aqua) to depolarize motoneurons and cause paralysis. Target site mutations were subsequently selected in resistant populations. (**B**) Biochemical effectors detoxify pyrethroids to nontoxic forms, e.g., phenoxybenzoic acid (PBAc), that are excreted (Figure 2). Pyrethroid is indicated in gold spheres; cytochrome P450 (CYP) proteins are indicated in green; carboxylesterases (COEs) are indicated in pink; rejuvenation of CYP (from blue oxidized to reduced green form) by nicotinamide adenine dinucleotide phosphate (NADPH) cytochrome P450-reductase (CPR) [45]. (**C**) Cuticular thickening and/or modifications are also features of resistance. Figure prepared using Biorender, with permission (Biorender.com, accessed on 22 March 2021). Chemical structures were drawn using Marvin JS (v. 17.4.3, chemaxon.com, accessed on 22 March 2021).

To protect motoneurons from the toxic effects of pyrethroids, mosquitoes have developed a full arsenal of metabolic and structural adaptations to detoxify or limit insecticide penetration (Figure 1). Examples of selected gene functional groups have been identified in genetic association, gene expression, and mechanistic studies [21,31,32,33,34,35,36], suggesting that multiple barriers and biochemical pathways contribute to heritable features of pyrethroid resistance. Still, specific intersections between these different pathways remain unexplored.

As contact insecticides, pyrethroids must first permeate the cuticular exoskeleton [18,19,20,21,22] and diffuse through the open circulatory system to reach targets on motoneurons. At initial points of contact, droplet size and the initial location of interaction with the exoskeleton can affect the toxicological response [37,38]. In resistant insects, following initial pyrethroid penetration, mixed function oxidases begin detoxification immediately. Rapid metabolic breakdown of pyrethroids is essential for insect survival. Insects with inadequate resources for detoxification succumb to paralysis and eventually death, due to pyrethroid binding to VGSC, which leads to neuronal depolarization [39]. Therefore, seemingly peripheral processes such as cell signaling, alterations to gene expression, and/or RNA processing may be key to augmentation of a swift resistance response, and therefore survival in resistant mosquitoes. For example, cell signaling amplifies the resistance response by stimulating expression of biochemical resistance effectors, e.g., cytochrome P 450 (CYP) genes [34]. Moreover, an open chromatin configuration is crucial to enhanced gene expression [40,41]; however, such studies have not been done in the context of mosquito insecticide resistance. At the post-transcriptional level, noncoding RNA regulation of gene expression may also condition resistance [42]. Unfortunately, most of these processes are largely understudied in mosquitoes, so the precise networks involved remain largely unknown.

Importantly, all of the processes outlined above occur prior to detoxification, except for cases in which pre-existing detoxification components are already present. Following detoxification, efflux pumps [43] may help excrete pyrethroid metabolic byproducts from cells and tissues. Moreover, Malpighian tubules also are likely to be important to elimination of breakdown products [44]. Taken together, these examples highlight a network of cellular processes that contribute toward pyrethroid resistance phenotypes. Assessing them in a systematic way could provide a way forward to the design of more effective insecticides with enhanced safety profiles.

## 2. Biochemical Resistance

### 2.1. Detoxification

**Figure 2:**
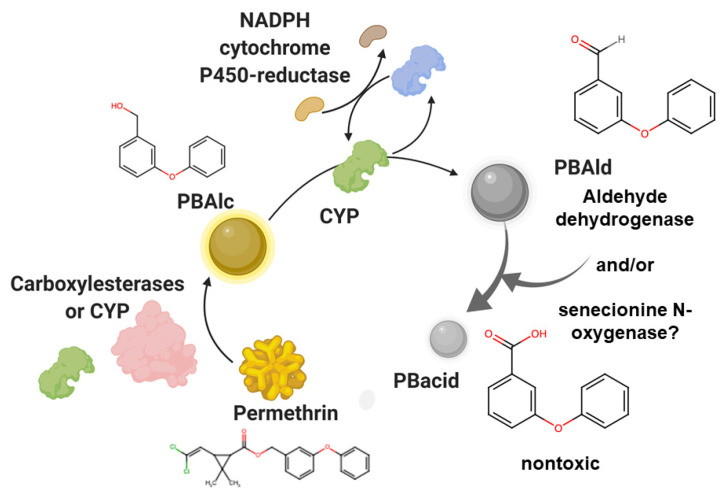
Pyrethroid detoxification cascade is mediated by carboxylesterases (COEs), cytochrome P450s (CYP6, CYP9) and other oxidation/reduction proteins. Permethrin is converted to PBAlc (phenoxybenzyl alcohol) by COEs and/or CYP9/CYP6 [45]. The metabolic intermediate PBAlc is converted to PBAld (3-phenoxybenzaldehyde). Aldehyde dehydrogenase is a demonstrated oxidizer of PBacid [54]. PBAc is nontoxic in larvae [45]. Recent genetic association evidence also implicated senecionine N-oxygenase as a possible player in pyrethroid resistance [31,89]. Proposed involvement of aldehyde dehydrogenase or senecionine N-oxygenase on right. Oxidized groups are shown in red in chemical structures. Figure prepared using Biorender, with permission (Biorender.com, accessed on 22 March 2021).

Pyrethroid detoxification is a multi-step process, producing several metabolic intermediates [19,45,46,47]. Specific pyrethroid detoxification mechanisms are often measured using biochemical assays of individual mosquitoes [48,49,50]. The current understanding of detoxification is as follows. CYPs and carboxylesterases (COEs) or other mixed function esterases [51] sequentially convert permethrin to metabolic intermediates and then to nontoxic moieties for subsequent excretion, perhaps through Malpighian tubules [44] (Tables S1 and S2). COEs convert permethrin to 3-phenoxybenzyl alcohol (PBAlc) [52,53]. Then, CYP orthologs convert PBAlc to 3-phenoxybenzaldehyde (PBAld). CYPs are also able to convert permethrin to 4’HO-permethrin [19]. CYPs are regenerated to their active state by nicotinamide adenine dinucleotide phosphate (NADPH) cytochrome P450 reductase (CPR, Figure 2) [45]. Finally, PBAld is converted to the nontoxic moiety phenoxybenzoic acid by aldehyde dehydrogenases [54]. Direct attack by CYPs on pyrethroids has also been reported; in this case, esterase is not required.

As major components of biochemical resistance, members of the CYP gene family [45,55,56] provide good illustrations of the ways in which independent evolutionary events have resulted in multiple genomic modifications across mosquito species [32,57,58] in a region-specific manner [59,60,61] (Tables S1 and S2). Such region-specific differences may be due to dissimilarities in selective pressure and mosquito genetic backgrounds [62]. In particular, an array of genomic modifications, ranging from gene duplication to selection at promoter regions to upstream signal transduction pathways, all occur at CYPs. Examples of each of these are detailed below.

Widespread gene duplication has occurred at CYPs and other detoxification genes [63]. This has resulted in multiple CYP paralogs in all major vector mosquito species and likely initially arose in response to DDT application in the early 20th century [33,62,63,64,65,66,67,68]. Strikingly, DDT application was so effective that *Aedes aegypti* was thought to have been cleared completely from most of Central and South America by the mid-1960s [66]. However, *Ae. aegypti* populations clearly rebounded and are now in full force in these regions. In recent years, detoxification as a major resistance mechanism has risen to the forefront of the field, and the roles played by CYPs have subsequently been cemented into the cast of metabolic resistance effectors [51,55]. More recently, in the context of practical applications, analysis of field populations in areas of insecticidal spraying and/or bed net use also revealed dramatic selection at CYPs in resistant populations [69,70]. 

Consistent with convergent evolution of insecticide resistance across vector species and genetic backgrounds, there are multiple ways in which CYP genes have evolved to respond to the toxic effects of pyrethroids. Smith et al. proposed that, in colonized *Aedes* mosquitoes, CYP9M5 and CYP9M6 were controlled at the transcriptional level rather than selected at the codon level [71]. Consistent with this hypothesis, multiple CYP genes are overexpressed in response to permethrin treatment [35,56,71,72]. Further, housefly CYP genes show polymorphisms in upstream regulatory motifs that have been identified in spinosad/permethrin-resistant insects [73]. 

Another common type of selective pressure occurs in gene coding regions. For example, nonsynonymous mutations in CYP genes were associated with permethrin resistance phenotypes in natural collections from South America and Africa [62,63] (Tables S1 and S2). This is also consistent with reports of genetic association data describing deltamethrin resistance in natural *Ae. aegypti* collections from Mexico [74], as well as *Ae. aegypti* with other genetic backgrounds [32,33,68]. Moreover, duplicated and high expression level CYP9M10 genotypes are present among multiple collections of *Culex quinquefasciatus* from Asia and Africa [75,76]. Similar results were also found for CYP6 orthologs in anophelines [57]. Fitness costs associated with selection for pyrethroid resistance should not be ignored when considering these effects. In *Anopheles funestus*, selection of CYP6P9a and glutathione S-transferases (GSTs), and the epsilon class in particular (GSTe), is associated with a reduction in fitness [77,78].

Functional studies have clarified roles of particular orthologs (CYP9, CYP6) and subclasses (e.g., CYP6P9b [70]) in permethrin detoxification [19,45,46]. One potential route of detoxification is outlined in Figure 2. Other studies have also implicated CYP6 and CYP9 subtypes at certain points in pyrethroid detoxification [45,62,64,79,80]. In one report, recombinant genes from *Cx. quinquefasciatus* in fall armyworm *Spodoptera frugiperda* cell culture provided support for the importance of CYP6AA7 and CYP9M10 in a portion of the detoxification cascade [45]. Similarly, aedine CYP9M6 and CYP6BB2 also participate in specific steps of detoxification [19].

The genomic modifications described above, whether due to gene duplication, altered promoters, or coding changes, all contribute toward an enhanced monooxygenase activity against pyrethroids. Such large increases in CYP activity could substantially increase the overall oxidative state of affected organisms. To mitigate these deleterious effects, redox components are needed to maintain cellular homeostasis. Consistent with this hypothesis, a number of redox proteins other than CYPs have also been implicated in metabolic resistance. For example, GSTs are in this category and therefore could help stabilize redox levels [51] (Tables S1 and S2). In particular, GSTe resistance alleles were identified in a number of *Anopheles* spp. [78,81,82,83,84,85]. GSTs protect insects from oxidative damage caused by permethrin-induced lipid peroxidation [86,87]. In addition, Kostaropoulos et al. showed that GSTs are able to bind directly to pyrethroids, suggesting a direct role in degradation [88]. Intriguingly, although CYPs and GSTs have undergone genomic modifications, other pathway components, e.g., CPR, the enzyme that reactivates CYPs, are present in single copy in anophelines. Therefore, reactivation of CYPs may represent a chokepoint in the detoxification pathway.

### 2.2. Cuticular Modifications

The exoskeleton provides the first barrier to insecticidal penetration. Thus, not surprisingly, modifications to cuticular components and cuticle thickening have been described in pyrethroid-resistant mosquitoes. Specifically, thickening of exoskeleton cuticular layers slows permethrin penetration in *Anopheles* spp. [18,21], *Ae. aegypti* [19], and non-vector insects [90,91]. One feature of exoskeleton thickening is an increase in the concentration of cuticular hydrocarbons (CHC). Notably, CYP4 genes have been implicated in CHC production in anophelines [18]. Differential localization of CYP4 paralogs is thought to contribute to the resistance response. For example, CYP4G17 is present throughout cellular cytoplasm of oenocytes (one of several types of hemocytes in the open circulatory system), consistent with localization to microsomes. In contrast, CYP4G16, which was specifically shown to produce CHCs, is localized to the cell periphery. More investigations are needed to fully understand the mechanisms associated with CYP4-mediated increases in CHC composition and their association with resistance.

### 2.3. Synergisms

Selection at VSGC target sites, combined with selection at a variety of CYP or other detoxification genes, substantially enhances permethrin resistance [92]. Specific mechanisms of interaction between CYPs and VGSC among different species have only recently been described due to the genomic complexities of outbred native mosquito populations. The use of the fruit fly model organism *Drosophila melanogaster* (*D. melanogaster*) and transgenic technology allowed Samantsidis et al. to look specifically at changes to permethrin resistance ratios, that is, the ratio of difference in lethal concentration 50% (LC50) rates between animals with PARA (VGSC V1016G) target site resistance alone versus those with target site mutations and also expressing *Aedes aegypti* CYP9J28 [92]. 

In studies of mosquito field populations, the combined effects of detoxification and target site sensitivity can be inexpensively teased apart, in a general way, using enzyme inhibitors, also known as synergists. These chemicals were used to implicate esterases and oxidases in pyrethroid resistance, due to the increased efficacy of pyrethroids when used in the presence of oxidase inhibitors, e.g., piperonyl butoxide (PBO) [93]. When used in combination with deltamethrin or permethrin, PBO abrogates apparent resistance due to the inhibition of oxidases, e.g., CYPs [94]. Another inhibitor, S,S,S-tributylphosphorotrithioate (tribufos), shows combined inhibitory effects on both oxidases and esterases [95]. Diethyl maleate (DEM) inhibits glutathione-S-transferase [96], as does etacrynic acid [97]. Lastly, triphenyl phosphate (TPP) is a COE inhibitor [98]. Because insecticide resistance has independently evolved in many mosquito populations, it is essential to evaluate specific mechanisms on a population-by-population basis, in order to determine the best approaches for pest management for a given location and species [99]. Such approaches have led to the successful use of synergists, alongside pyrethroids, in long-lasting insecticidal nets (LLINs) in some regions of Africa, to reduce the incidence of malaria [99,100]. Many ultra-low volume spraying formulations intended to control *Ae. aegypti* or *Cx.* spp. also include PBO to synergize pyrethroids [101].

### 2.4. Excretion of Metabolic Intermediates

ABC transporters are a very large class of transmembrane proteins that have been extensively tied to insecticide resistance in a variety of invertebrates. Gene duplication and increased expression of multiple subtypes of ABC transporters (ABCB, ABCC, ABCG) have been reported in response to pyrethroid exposure [102,103,104,105,106]. Transporters in this class are predicted to act as efflux pumps that clear pyrethroids or toxic intermediates from cells. ABCG proteins are expressed in a variety of mosquito tissues [107]. It is currently unclear whether mosquito ABC transporter orthologs contribute toward the penetration barrier of the exoskeleton, as occurs with ABCH transporters in drosophilids [108], whether they function primarily to excrete metabolic intermediates at the cellular level, or whether various paralogs participate in different aspects of excretion.

## 3. Newly-Described Features of Resistance

### 3.1. Post-Transcriptional Regulation

#### 3.1.1. Small RNA Profiling

Small noncoding RNAs (sRNAs), specifically microRNAs (miRNAs), regulate gene expression via miRNA interference (miRNAi) to control a wide array of cellular metabolic processes. Gene expression control can either occur through stalling of protein translation or the mRNA decay pathway (reviewed in [109]). Seong et al. used selected *D. melanogaster* DDT-resistant and susceptible strains to explore the roles of sRNAs in regulation of metabolic resistance genes and determine whether differential sRNA and transcript levels were associated with resistance [42]. They found that lower levels of key miRNAs were associated with enriched CYP target transcript levels in a DDT-resistant strain compared to a comparable susceptible strain. Because miRNAs (miRs) typically reduce target gene expression, reduced miRNA levels are expected to occur in the presence of CYP expression, which would lead to increased detoxification. Seong et al. concluded that reduced levels of members of the miR-310 gene cluster, which expresses miR-311, miR-312, and miR-313, occurred concomitantly with enrichment of CYP transcript levels. Such studies have been instrumental in opening new doors to future exploration of the nuances of post-transcriptional gene regulation in insecticide resistance. Much more needs to be done to understand the mechanisms involved.

Similar studies have explored comparable relationships between miRNA regulation, resistance effector gene expression, and pyrethroid resistance in mosquitoes. For example, sRNA profiling was done in deltamethrin-selected and susceptible *Cx. pipiens* lines to identify differentially-expressed miRNAs and putative miRNA targets associated with resistance phenotypes [110]. A study of *Cx. pipiens pallens* found that miR-13664 regulates expression of CYP314A1 [111]. Lastly, a study by Tian et al. showed the somewhat surprising result that reduced levels of CYP6N23 were correlated with increased pyrethroid resistance in *Cx. p. pallens* via miRNA-mediated post-transcriptional regulation [112]. This is in marked contrast to the many reports that have described the positive correlation between CYP6 and other CYP paralogs with pyrethroid resistance, once again underscoring the need to validate putative resistance effectors in relevant genetic backgrounds before drawing conclusions about possible roles in resistance.

#### 3.1.2. Nuanced Post-Transcriptional Regulation of VGSC

Polymorphisms in miRNA genes could alter the breadth of regulatory control targets or differentially control expression of metabolic resistance genes. Specifically, structural variation in the precursors, or pre-miRNAs, could provide a way to modulate post-transcriptional regulation in response to the toxic effects of permethrin exposure and mitigate fitness cost. In particular, different pre-miRNA structural configurations could hasten or slow miRNA processing [113], which would, in turn, slow the rate of downstream post-transcriptional regulation of gene expression. In this context, a polymorphism in the pre-miRNA for miR-33 was associated with the permethrin resistance phenotype in *Ae. aegypti* [114]. The pre-miR-33 polymorphism occurred in a region adjacent to the mature miRNA and therefore was not predicted to alter the mature miRNA sequence. Examination of the effects of miRNA overexpression indicated that miR-33 regulated VGSC transcript and protein levels. Expression of the pyrethroid target, VGSC, is also regulated post-transcriptionally by mRNA decay effector, PUMILIO, in a synaptic activity responsive manner [115]. 

In summary, reduction of the density of VGSC proteins on motoneuronal cell surfaces could give the mosquito’s metabolic system more time for detoxification effectors to clear pyrethroids and/or metabolic intermediates prior to irreversible toxic effects. Specifically, if fewer VGSC proteins were present on neurons, fewer toxin binding sites would be bound by pyrethroids. Consistent with this, a permethrin-resistant *Ae. aegypti* collection showed reduced VGSC protein levels compared to a genetically related susceptible strain [114]. The presence of fewer permethrin binding sites would prevent lethal effects while the host focused metabolic energy on detoxification and efflux of metabolic intermediates. 

### 3.2. New Potential Detoxification Effectors

Due to the wide variety of oxidation/reduction components identified in genetic association and RNA-seq studies, it is possible that other, yet undescribed, pyrethroid breakdown mechanisms are present in mosquitoes. For example, study of specific pyrethroid resistance effectors in heterologous insect cell culture systems revealed the retention of low levels of permethrin metabolic intermediate breakdown in the absence of heterologous expression of the *Cx. quinquefasciatus* major effectors CYP9 and CYP6 [45]. This evidence hinted at the idea that additional detoxification or redox homeostasis partners remain unidentified. Evidence supportive of a new, as yet uncharacterized, potential detoxification effector was identified in permethrin-treated *Ae. aegypti* collections. Specifically, genetic association analysis of knockdown-sensitive and resistant mosquito pools implicated senecionine N-oxygenase (SNO) in permethrin resistance [31]. Indeed, four SNO orthologs were present in the top 30% list of pyrethroid resistance genes. In other insects, SNO has been described as a pyrrolizidine alkaloid-specific oxidase [116], however it also acts on phenolic ring compounds (reviewed in [89]), which are present in the permethrin structure. Given the presence of multiple senecionine N-oxygenase orthologs in the high-association dataset, we hypothesize that hydroxylation of permethrin breakdown intermediates could be mediated by SNO. Clearly, any participation of SNO in permethrin detoxification must be validated in functional studies to substantiate this idea. Another potential example is sulfotransferase, which was identified in a transcriptomic analysis of deltamethrin-treated *An. gambiae sensu stricto* [35] but has not been characterized further.

### 3.3. Signaling Mechanisms

The presence of a broad array of effectors with association to knockdown resistance or lethality phenotypes is consistent with the hypothesis that mosquitoes mount a global signaling response to pyrethroid exposure that involves several biological pathways. In those insects that are unable to detoxify pyrethroids prior to penetration of motoneurons, the toxins subsequently bind VGSC proteins on motoneurons, leading to depolarization, paralysis, and possibly death [39]. Therefore, alteration of signal transduction cascades may provide a selective advantage with the enhancement of detoxification enzyme production and thus prevent lethal toxic effects of pyrethroids. For this reason, the study of upstream signal transduction events could reveal new options for enhancing current pyrethroids. Transcriptomic and genetic association studies have identified signaling genes [31,32,74]; however, very few have focused exclusively on the specific roles of signaling messengers [34,117].

G-protein coupled receptor (GPCR) cell signaling pathways typically involve GTP hydrolysis and activation of small GTPases, e.g., Ras, Rho, or Rab, for subsequent activation of second messenger systems that regulate gene expression and post-translational modifications of cellular constituents. In *Cx. quinquefasciatus*, GPCR activation of adenylate cyclase is expected to activate cyclic adenosine-monophosphate (cAMP), which is translocated to the nucleus for subsequent transcriptional activation of CYP genes [34]. Li et al. described a rhodopsin-like GPCR in *Cx. quinquefasciatus*, which, when transfected into *D. melanogaster*, increased permethrin tolerance by twofold. It is surprising that the addition of a receptor alone could make such a difference in a non-vector insect.

**Figure 3:**
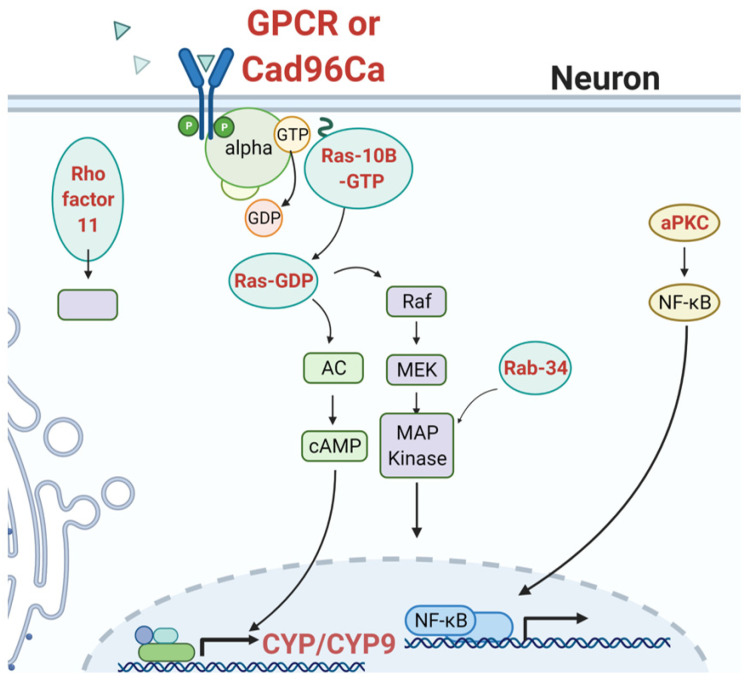
Signaling mechanisms contribute to pyrethroid resistance. Activation of the signaling mechanism could occur as part of the wound response [118,119,123]. GPCR signaling and subsequent upregulation of detoxification enzyme expression has been described in *Culex* spp. [34,117]. Increased resistance was observed in the presence of elevated cyclic adenosine-monophosphate (cAMP). Effectors in red font are genetically associated with pyrethroid resistance in natural *Ae. aegypti* collections from Mexico [31]. Atypical protein kinase C (aPKC), which helps maintain cell polarity, could activate NF-κb upregulation of resistance genes [122]. Figure prepared using Biorender, with permission (Biorender.com, accessed on 22 March 2021).

The activation signal for insecticide resistance-associated stimulation of the GPCR pathway is not known, however it could be a general stress response. This general hypothesis was formed from analysis of pyrethroid resistance genetic association data from natural *Ae. aegypti* populations [31]. For example, the polarized cell-specific cadherin (CAD96ca, tyrosine receptor kinase) [118] could serve as a damage sensor of excess calcium ions released upon pyrethroid-mediated neuronal depolarization, given that it is the signal transducer of wound-response genes in drosophilids [119] (Figure 3). Activation of CAD96ca leads to extracellular signal related kinases (ERK)-mediated signal transduction and transcriptional activation of wound-repair sites, via a positive feedback loop initiated by the transcriptional activator GRAINYHEAD (GRH) [120], which has also shown genetic association to pyrethroid resistance [31]. Atypical protein kinase C (aPKC) is also a target of GRH [121]. The gene product aPKC participates in the maintenance of cell polarity and signal transduction in polarized cells (reviewed in Shieh, 2002 [122]) (Figure 3). GRH is a conserved transcription factor that is present in all metazoans [123]. We speculate that GRH, aPKC, and CAD96a participate in the response to insecticide treatment by initiating a general stress response.

## 4. Convergent Evolution of Insecticide Resistance

Mosquito insecticide resistance studies have implicated gene orthologs in specific mosquito collections that do not occur in other collections [124]. For example, areas with hyperendemic dengue fever transmission subsequently had the highest rates of adulticidal spraying with subsequently higher insecticidal resistance rates compared to other locations [125]. Given that mosquitoes exist in reproductively isolated areas with limitations to gene flow [57,126,127,128,129], insecticide resistance has clearly evolved independently and over multiple iterations among global populations. The independent selection of knockdown resistance traits has also been described in field-collected housefly strains [130]. For this reason, we expect that specific genetic associations will be found in some populations but not in others. Nevertheless, there are similarities across species in the epistatic relationships or biochemical pathways that respond to insecticide treatment. 

## 5. Conclusions

The variety of resistance mechanisms described here are consistent with evolutionary selection on a variety of cellular maintenance and functional pathways across multiple species of pyrethroid resistant mosquitoes, with selection of unique effectors in reproductively isolated populations. Recent insights into transcriptional regulators provide promise for future identification of chokepoints or bottlenecks wherein novel targets of insecticides might be identified. Though great strides have been made to better describe the mechanistic features of pyrethroid resistance in recent years, most studies still rely on double-stranded RNA gene knockdown rather on generation of transgenic models. Moreover, the roles played by alternative transcriptional isoforms, and post-transcriptional and post-translational alterations to resistance effector proteins, as related to pyrethroid resistance, have not been described in mosquitoes. More detailed study in these areas is needed, which could lead to development of new insecticidal targets with enhanced effectiveness over existing pyrethroid formulations and prevent the transition to more dangerous or environmentally harmful insecticides.

## 6. Criteria for Review 

The PubMed database was searched using the search terms “pyrethroid resistance mechanism” with inclusive dates 2010 to 2020. Of 1031 original articles with Pubmed ID numbers, individual articles were searched for relevant terms relating to mosquito metabolic resistance, using Bibliometrix (R, version 3.0.0); results are presented in Tables S1 and S2.

## Data Availability

No new data were created or analyzed in this study. Data sharing is not applicable to this article.

## Supplementary Materials

The following are available online at https://www.mdpi.com/2075-4450/12/4/276/s1, Table S1 (*Anopheles* Resistance Genes) and Table S2 (*Aedes* and *Culex* Resistance Genes) show the articles covered by the review criteria, along with the specific pyrethroids that have been reported for each. A part of references are cited in the Supplementary Materials.
