# Supplementary material for: From Global to Local—New Insights into Features of Pyrethroid Detoxification in Vector Mosquitoes
Source: Insects. 2021 Mar 24;12(4):276. doi: 10.3390/insects12040276 (PMC8063960; doi:10.3390/insects12040276)
Supplement: Supplementary file 1 [file insects-12-00276-s001.pdf]

Table S1 *Anopheles* Resistance Genes

Metabolic resistance

Species	gene	Reference PMID	Pyrethroid
Anopheles spp.			
An. albimanus	CYP4C26[131] CYP9K1[131] CYP6P5[131]	30699158	alpha-cypermethrin deltamethrin
An. arabiensis	GSTe4 [15], CYP6M2 [15], CYP6P3[15]	24886129	NA
	CYP4G16[132]	24314005	lambda-cyhalothrin
	CYP6P4[133]	23299100	permethrin deltamethrin
	CYP9L1[134]	22676389	permethrin deltamethrin
An. coluzzi	CYP, general[135, 136]	31796068; 31126311	permethrin deltamethrin α-cypermethrin
	CYP6Z1[137]	29618373	permethrin
	CYP9K1[56, 137]	29674455; 29618373	permethrin deltamethrin
An. funestus	CYP, general[81, 138-140]	30402485; 2753112 25333491; 32331386	permethrin deltamethrin
	CYP6M7[62, 124, 141-144]	27628765; 29115954; 28003461; 27519696; 25261072; 22948188	permethrin deltamethrin
	CYP6P9a/b[16, 62, 64, 69, 70, 83, 124, 141, 143, 145-149]	30458849; 28428243; 27628765; 26370361; 22110757; 31604938; 30923819; 29115954; 28003461; 27135886; 25261072; 23248325; 20686697	permethrin deltamethrin lambda-cyhalothrin bifenthrin
	CYP6Z1[142, 146]	27135886; 22948188	permethrin deltamethrin

	CYP6Z3[142]	22948188	NA
	GST[81, 147]	20686697; 32331386	permethrin
	GSTd1-5[83], GSTD3[83]	30458849	permethrin
	GSTe2[16, 83, 124, 138, 142, 148, 150]	27628765; 27531125; 26370361; 30458849; 30433868; 28428243; 22948188	permethrin deltamethrin lambda-cyhalothrin
	Alpha-esterases[140]	30402485	permethrin deltamethrin
An. gambiae	CYP, general[55, 151]	23189131	permethrin deltamethrin
	CYP4G16[22, 59, 152]	32156617; 31554225; 28894186	permethrin deltamethrin etofenprox
	CYP6M2[22, 46, 59, 143, 153-156]	31315630; 31554225; 29587635; 29115954; 28894186; 24651294; 21324359	permethrin deltamethrin etofenprox α-cypermethrin
	CYP6P1[143]	29115954	deltamethrin
	CYP6P3[157]	28894186; 24651294; 23380570	permethrin deltamethrin lambda-cyhalothrin etofenprox
	CYP6P4[20, 59]	31554225; 30740870	permethrin deltamethrin
	CYP6P9a[57, 60]	30894503; 28151952	NA
	CYP6Z1[59, 146, 152]	32156617; 31554225; 27135886	permethrin deltamethrin
	CYP6Z2[157]	23380570	permethrin deltamethrin lambda-cyhalothrin
	CCYP9K1[59]	31554225	permethrin deltamethrin
	GSTd3[20, 153, 155]	31315630; 30740870; 29587635	permethrin deltamethrin α-cypermethrin

	GSTe2[22, 143]	28894186; 29115954	permethrin<br>deltamethrin<br>etofenprox
	GST[151, 158-160]	24358177; 23641777; 23189131; 22472088	permethrin<br>deltamethrin<br>etofenprox<br>α-cypermethrin<br>lambda-cyhalothrin
	GSTS1-2[143]	29115954	deltamethrin
	Mixed function oxidases[158-160]	24358177; 23641777; 22472088	permethrin<br>deltamethrin<br>etofenprox<br>α-cypermethrin
	esterases[151, 158-160]	24358177; 23641777; 23189131; 22472088	permethrin<br>deltamethrin<br>etofenprox<br>α-cypermethrin<br>lambda-cyhalothrin
An. stephensi	GST[161, 162]	30584554; 24947216	cyfluthrin<br>alphamethrin<br>benfluthrin
	Alpha-esterases[161]	30584554	cyfluthrin
An. sinensis	CYP[163, 164], GST[163, 164]	25499700; 23405157	deltamethrin
	CYP6Z2[165], CYP6P3v1[165], CYP6P3v2[165], CYP9J5[165] and CYP306A1[165]	29393554	NA
Anopheles, mixed species	GST, general[84, 97, 166-170]	30509288; 29566682 29310704; 27030033; 26296644; 25213601; 27378358	permethrin<br>deltamethrin<br>etofenprox<br>α-cypermethrin<br>lambda-cyhalothrin
	esterase[169, 170]	26296644; 27378358	deltamethrin<br>lambda-cyhalothrin
	CYP, general[59, 168]	27030033; 31554225	permethrin<br>deltamethrin

	CYP6P3[170]	27378358	deltamethrin
	CYP6M2[170]	27378358	deltamethrin
	GSTD3[170]	27378358	deltamethrin
	Mixed-function oxidases[167]	29310704	permethrin deltamethrin α-cypermethrin lambda-cyhalothrin

Table S2 *Aedes* and *Culex* Resistance Genes

Species	Metabolic resistance gene	Reference PMID	Pyrethroid
Culex spp.			
Culex pipiens	CYP9M10[171]	25987223	See review
Culex pipiens pipiens	CYP[172]	30766584	deltamethrin
Culex pipiens pallens	CYP314A1[111]	29966536	deltamethrin
	CYP6AA9[173, 174]	26377942; 25880395	deltamethrin
	CYP9AL1[175]	30904950	deltamethrin
	CYP9M10[176], Gstd1[176], Gstd2[176]	29107250	cypermethrin
Culex quinquefasciatus	CYP9M10[14, 75, 76, 177, 178]	27095599; 23632895; 24155662; 21858101; 21540111	permethrin
	CYP325K3v1[178], CYP4D42v2, CYP9J45, CYP325G4, CYP4C38, CYP4H40; CYP6Z12, CYP9J33, CYP9J43, CYP9J34, CYP306A1,CYP6Z15, CYP9J45, CYPPAL1, CYP4C52v1, CYP9J39	24155662	permethrin
	CYP6AA7[76], CYP9J40, CYP9J34	21858101	permethrin

Aedes aegypti	ABCB4[102]	22720108	NA
	GST, general[8, 25, 51, 179-184]	30552882; 26307496; 25454522; 21272394; 21394876; 21226942; 19556575; 29432488; 24299217	deltamethrin permethrin λ-cyhalothrin cypermethrin cyfluthrin
	Carboxylesterase (COE)[8, 17, 31, 185, 186]	28187780; 30758862; 28114328; 26588076; 24299217	deltamethrin permethrin
	GST[8, 31, 185, 187]	30758862; 31175834; 28114328; 24299217	permethrin deltamethrin cypermethrin
	GSTe2[186]	28187780	deltamethrin
	CYP[32, 74, 102, 188, 189]	31519246; 30758862; 24299217; 24593293; 22720108	permethrin deltamethrin (1R)-trans-fenfluthrin etofenprox cyfluthrin bioallethrin cypermethrin fenpropathrin
	CYP4C50[71]	30452436	permethrin
	CYP6BB2	28187780; 30452436; 26588076; 24945250	deltamethrin permethrin
	CYP6CB1[185]	28114328	NA
	CYP6F2/3[71]	30452436	permethrin
	CYP6M11[17, 186]	28187780; 26588076	deltamethrin
	CYP6N12[17]	26588076	deltamethrin
	CYP6Z8[71]	30452436	permethrin

	CYP9J9[17]	26588076	deltamethrin
	CYP9J10[17, 190]	31027587; 26588076	deltamethrin
	CYP9J19[190]	31027587	deltamethrin
	CYP9J23[186]	28187780	deltamethrin
	CYP9J26[102, 185]	28114328; 22720108	NA
	CYP9J27[185]	28114328	NA
	CYP9J28[190]	31027587; 28347352	deltamethrin
	CYP9J32[13]	21324051	deltamethrin
	CYP9M4[185]	28114328	NA
	CYP9M5[71]	30452436	permethrin
	CYP9M6[19, 71]	30452436; 24945250	permethrin
	Alpha- and beta-esterases[25, 179, 182, 191]	26307496; 25454522; 23540124; 21272394	deltamethrin permethrin λ-cyhalothrin cypermethrin cyfluthrin
	Esterases[51, 180, 183, 184]	21394876; 21226942; 19556575; 29432488	Permethrin deltamethrin λ-cyhalothrin cypermethrin
	Mixed function oxidases[25, 179, 182]	26307496; 25454522; 21272394	deltamethrin permethrin λ-cyhalothrin cypermethrin cyfluthrin
Ae. albopictus	GST, general[181, 192, 193]	29298700; 30418967	deltamethrin
	GSTS1/3[194]	27094778	permethrin
	CYP, general[181, 193]	29298700; 30418967	deltamethrin

	CYP6A8[193]	30418967	deltamethrin
	CYP6P12[194]	27094778	permethrin
	esterase[193]	30418967	deltamethrin
	COE, general[192]	29298700	deltamethrin
